# Collaboration between Public Health and Schools: An Example of an Integrated Community Social Care Model

**DOI:** 10.5334/ijic.7529

**Published:** 2023-08-18

**Authors:** Audrée Jeanne Beaudoin, Marilyn Gagnon, Mathieu Roy, Irma Clapperton, Annie Lambert, Emmanuelle Jasmin, Edwige Ducreux, Annie Desrosiers, Claudine Martin

**Affiliations:** 1 Institut universitaire de première ligne en santé et services sociaux, CIUSSS de l’Estrie – CHUS, CA; 2 Faculté de médecine et des sciences de la santé, Université de Sherbrooke, CA; 3 Direction de santé publique, CIUSSS de l’Estrie – CHUS, CA; 4 Faculté des lettres et des sciences humaines, Université de Sherbrooke, CA; 5 INSPÉ Lille Haut de France, FR

**Keywords:** mental health, health promotion, case study, intersectorality, proximity, Santé mentale, promotion de la santé, étude de cas, intersectorialité, proximité

## Abstract

**Introduction:**

There is a need to improve public health interventions to promote youth social and emotional development in close collaboration with schools, families and local communities. A close intersectoral collaboration between the regional public health, schools and school boards was established to co-construct and implement “Positive Intervention (PI)” in the Eastern Townships region (Quebec, Canada). This paper describes its implementation according to the “Integrated Community Care (ICC)” framework.

**Description:**

PI is a collaborative and personalized intervention leaning toward an integrated community social care model. In fact, PI relies on the close proximity between Public Health and their educational counterpart as well as their individual temporality. However, PI offered mainly social services and its relationships with Primary Care services was not yet a priority.

**Discussion:**

The results show that it is possible to develop and implement an intervention promoting positive mental health in children, with and for local organisations. The level of integration between schools and Public Health services achieved after only 6 months of implementation is encouraging.

**Conclusion:**

More research is needed to thoroughly document the implementation, social validity, and effects of such an intervention by taking in the point of view of all stakeholders.

## Abstract (fr)

**Introduction:**

Il est nécessaire d’améliorer les interventions de santé publique pour promouvoir le développement social et émotionnel des jeunes en étroite collaboration avec les écoles, les familles et les communautés locales. Une telle collaboration intersectorielle entre la santé publique régionale, les écoles et les commissions scolaires a été mise en place pour coconstruire et mettre en œuvre l’Intervention positive (IP) en Estrie (Québec, Canada). Cet article décrit sa mise en œuvre initiale selon le cadre conceptuel des soins de santé et services sociaux intégrés en proximité des communautés.

**Description:**

L’IP est une intervention collaborative et personnalisée qui s’inscrit dans un modèle de services sociaux intégrés en proximité des communautés. En fait, l’IP s’appuie sur une forte proximité entre les services de santé publique et les milieux scolaires, ainsi qu’un ajustement à la temporalité des partenaires. Cependant, l’IP offre principalement des services sociaux et ses relations avec les services de soins primaires n’étaient pas encore une priorité.

**Discussion:**

Les résultats montrent qu’il est possible de développer et de mettre en œuvre une intervention de promotion de la santé mentale positive chez les enfants, avec et pour les organisations locales. Le niveau d’intégration entre les écoles et les services de santé publique atteint après seulement six mois de mise en œuvre est encourageant.

**Conclusion:**

Des recherches supplémentaires sont nécessaires pour documenter de manière approfondie la mise en œuvre, la validité sociale et les effets d’une telle intervention en prenant en compte le point de vue de toutes les parties prenantes.

## (1) Introduction

Early childhood is the most important developmental stage as it sets a critical foundation for the life course [1]. However, Rimm-Kaufman et al. reported that 20% of kindergarten teachers indicated that at least half of the children in their class had insufficient social and emotional skills to function adequately in school [2]. Moreover, according to a Canadian study, 10.2% of children were considered vulnerable in their social skills development and 11.5% in relation to emotional maturity when they entered school [3]. It is crucial to act proactively to promote positive mental health in children and to prevent social and emotional difficulties associated with behavioral problems and mental health disorders in adolescence and adulthood [4,5]. In addition, many children who are not considered at risk may still need mental health interventions, because of stressful situations or other risk factors to which they are exposed [6]. A growing body of evidence suggests that a public health approach is needed to promote positive mental health in children [5]. Mental health is more than the absence of mental disorders. In this sense, positive mental health refers to the positive aspects of human behavior and adaptation to different situations [7,8], including emotional regulation and social skills [9].

Given that children’s mental health is closely connected to their social environment (family, friends, school, and community) [10,11], it is important to focus on improving the quality of their socioeducational environment, including the home, school, and the community, in order to promote the development of children’s social and emotional skills [12,13,14]. The World Health Report recognized that “schools are crucial in preparing children for life, but they need to be more involved in fostering healthy social and emotional development” [8]. Since the 2000s, there has been a broad movement toward expanded school mental health programs, notably in the United States [6]. A few programs aiming at promoting mental health have been developed and implemented in schools in recent decades [15,16,17,18,19]. However, it is important to work collaboratively across all environments in which children evolve, as stated in the Ottawa Charter for Health Promotion [20]. Lister-Sharp et al.’s review reported that interventions which include changes to the school environment, and which promote family and local community’s involvement are more likely to be effective [21]. One of the main challenges is to bring organizations from different sectors together in a common pursuit of positive mental health [22].

Thus, there was a need in public health services in the “Centre integré universitaire de santé et de services sociaux de l’Estrie – Centre hospitalier universitaire de Sherbrooke (CIUSSSE-CHUS)” (Québec, Canada) to promote youth social and emotional development in close collaboration with school, families and local communities. To that end, a major reorganization of services fostering children’s healthy social and emotional development was initiated. A collaborative approach with intersectoral partners (Public Health, Education [school boards] and parents) explored their needs as well as the best socially acceptable public health practises, and mobilized all partners towards a shared and unifying change, namely the adoption of “Positive Intervention (PI)”. This approach aims at promoting children’s social and emotional skills by using strategies promoting positive mental health. A close intersectoral collaboration was established between the Regional Public Health, schools, and school boards to co-construct and personalize the intervention. PI, inspired by the principles of positive psychology, has never been documented, and its great flexibility can result in variability in its implementation. Considering that this intervention model is co-constructed and implemented with and in local communities, this paper aims to describe the implementation of PI carried out jointly by the regional Public Health organization and elementary schools according to the “Integrated Community Care (ICC)” framework. This is a first step in assessing the feasibility of this new and innovative service offered to children aged 4 to 8 years.

## (2) Methods

### (2.1) Design

A concurrent mixed methods design was used to describe the initial implementation of PI [23]. First, a quantitative descriptive phase aimed to broadly describe the implementation of the intervention model. Simultaneously, an in-depth qualitative phase was conducted with three schools representing different contexts to detail the implementation of said model.

An advisory committee including researchers, Public health professionals, parents, and school board administrators met regularly to co-develop the research project, to provide critical input and to ensure shared decision-making with all stakeholders [24,25]. The research project has received ethical approval from the Research Ethics Board of the CIUSSSE-CHUS.

### (2.2) Study population

The target population for this research was public elementary schools on the territory of the CIUSSSE-CHUS. Each participating school had to: 1) be a public elementary school, 2) be located in the CIUSSSE-CHUS territory, and 3) agree to receive PI services. Quantitative data were collected for all eligible schools. Qualitative data were gathered within three schools (n = 3 cases) chosen according to criteria selected by the advisory committee:

- affiliated school board (three different school boards out of the five on the territory),
- language of instruction (one English and two French schools),
- socioeconomic level (at least one school considered disadvantaged [deciles 8, 9 or 10] according to the socio-economic environment index [26]), and
- rurality (at least one rural and one urban school).

Based on these criteria, the advisory committee suggested three schools that would provide the desired representativeness. School board managers on the advisory committee first contacted these schools to explore their interest. The research team then presented the research project in greater detail and obtained the principal’s consent for each of the three targeted schools. Finally, informed consent was obtained from all participants prior to interviews.

### (2.3) Data collection and analysis

This research project was conducted simultaneously with the implementation of PI. The quantitative data collection was carried out using clinical administrative data compiled by Public Health professionals during their interventions, as well as a questionnaire (Request for support; see supplementary material) created by the Public Health department and completed by schools wishing to collaborate with them. When completing support requests, schools were asked to indicate whether or not they agreed to share their information with the research team. Consent was obtained for 46 of the 48 support requests (96%) received. Out of the two schools not included in the analysis, one did not reply to the e-mails sent out by the research team, and the other preferred not to participate due to a change of leadership within the school during the school year. Descriptive analyses were performed on categorical data (frequency and percentage) and numerical data (mean and standard deviation) from the general data collection.

The qualitative data were collected to describe in detail the contexts and support requests of the three cases. To do so, individual interviews were conducted, followed by focus groups. Interviews were conducted with school board managers (n = 3), school system navigators (n = 3), school principals (n = 3), and public health professionals (n = 3) involved with the targeted school. Subsequently, four focus groups were conducted: one group included school board (n = 3) and Public Health (n = 1) managers, whereas the other three groups (one per targeted school) were held with the assigned health care professional, the school principal, the school system navigator and staff members. Interview and focus group guides were created by the research team and the advisory committee to ensure that the questions would focus on the implementation process of PI and on the contextual factors that explain the differences between schools. Data analysis was conducted by two team members using the *QDA miner* software. Descriptive codes were inductively developed by a team member (MG) and verified by a second team member (AJB). Regular meetings among coders were held to review newly developed codes, deepen coders reflexivity, and make iterative modifications to the coding tree. A second cycle of coding was conducted via QDA Miner to refine codes and identify overarching categories. After coding each case, intra- and inter-case analyses were performed for the three schools [27].

Finally, quantitative and qualitative data were integrated using the Conceptual Framework for ICC [28] to highlight strengths, limitations, and suggestions to further improve PI’s implementation.

### (2.4) Conceptual framework

This paper is based on the conceptual framework for ICC [28]. As defined by Thiam et al., ICC is “an interweaving of localized and temporalized health care and social care interventions provided in proximity (spatial and relational) in an interdisciplinary and cross-sectoral way. ICC aims to improve physical and mental health, well-being and empowerment, as well as facilitate access and use of care, particularly among disadvantaged populations or those not served by the health and social care system” [28]. To do so, ICC “must not only deliver primary care in proximity to the population, but act upstream on the social determinants of health” [28].

### (2.5) Intervention

In this section, PI is described according to the analysis parameters of ICC by Thiam and colleagues, namely the setting, the targeted population, the objectives pursued, and the approach used [28].

#### (2.5.1) Setting

PI aims to provide proximity services that promote positive mental health for children. Elementary schools received joint support and coaching from public health professionals (i.e., social workers or psychoeducators) and school system navigators (i.e., educational consultants or psychoeducators). The school system navigators’ mandate is to facilitate the planning and implementation of strategies in collaboration with the school community and to follow up on the actions taken.

#### (2.5.2) Targeted population

All children aged 4 to 8 years attending an elementary public school in the Eastern Townships region (Quebec, Canada) were targeted. In order to ensure health equity, special attention was paid to include schools from low socio-economic levels, based on the provincial socio-economic environment index. This index is calculated for each school according to the proportion of mothers without degrees and households without jobs [26]. In addition, PI’s universal approach allows every child to benefit from a supportive environment promoting their social and emotional development.

#### (2.5.3) Objectives pursued

PI promotes positive mental health and the development of social and emotional skills in children. It is based on interactions between students and adults and combines caring, empathy, and positive discipline [29]. These elements are considered a necessary foundation for the creation of a healthy and strong attachment bond between children and significant adults such as parents, teachers, and school personnel. A good attachment bond supports the child’s social and emotional development and promotes academic success. PI acknowledges the immaturity of children’s brains and their need for significant adults to help them deal with emotions and feelings. These positive emotional experiences in turn fuel the brain’s maturation process and can improve attachment. Finally, children develop kindness and empathy towards others by being in contact with caring and empathetic adults, which fosters better quality of social interactions [30].

#### (2.5.4) Approach used

PI involves a co-construction of strategies with different partners (e.g., schools, families, and local community) according to the needs and contexts. First, a global and multifactorial analysis of the school’s needs is used to jointly determine the main objectives. In each school, this was done through discussions involving at least the health care professional, the school principal, and the school system navigator. Depending on the school context and needs, other school staff members can be included in this initial phase. Strategies are then planned and carried out in collaboration with the school personnel (e.g., administrators, teachers, supervisors, daycare educators, complementary services providers, school transportation staff and others). This co-developed plan of action must then include one or more of the following activities in the school: awareness-raising activities, skill building activities for staff members, observation in the environment, coaching of staff members, development of workshops for parents, activities in the classroom, and sharing educational tools. Public health professionals then act as coach to foster empathetic and caring environments which promote feelings of security and attachment in students.

**Figure 1:**
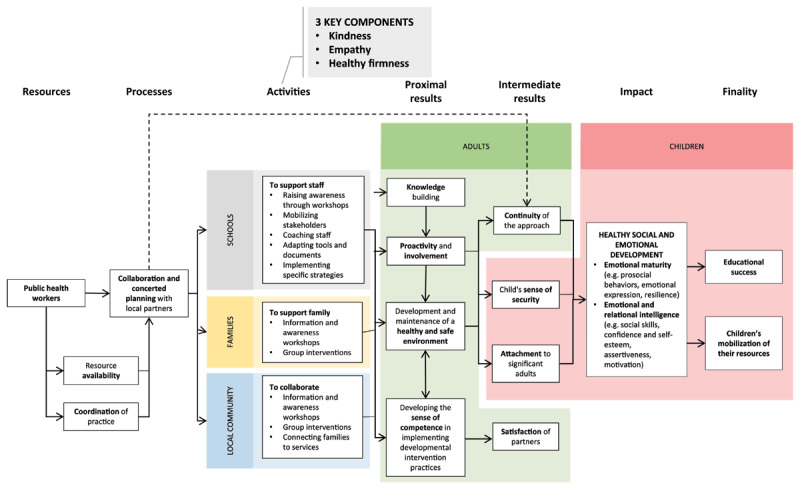
Logical model of the Positive Intervention.

As illustrated in the logical model (Figure 1), strategies were implemented by public health professionals of the CIUSSSE-CHUS to indirectly benefit students aged 4 to 8. Building on adults’ strengths, PI primarily targets the development of knowledge and skills. In this way, adults are actively involved in creating a safe and healthy environment for children. In doing so, PI seeks to promote children’s healthy social and emotional development, educational and personal success, as well as relational engagement.

## (3) Results

### (3.1) Quantitative data: Overall description of the implementation of PI

Between September 2019 and March 2020 (when schools were shutdown because of the pandemic), 46 schools had requested support and agreed to participate in the research project. The average socioeconomic background index (in deciles) for these schools was 7.09 (SD: 2.54). The most common strategies prioritized by schools to promote mental health were staff coaching (n = 43, 93%), awareness workshops (n = 36, 78%) and staff mobilization (n = 34, 74%). School personnel most often identified in requests for support were supervisors (n = 39, 85%), school principals (n = 39, 85%), daycare educators (n = 38, 83%), teachers (n = 27, 59%), and student support staff (n = 26, 57%). Overall, the support requests indirectly targeted approximately 12,065 children, including 5,527 children aged 4 to 8 years.

### (3.2) Qualitative data: Description of the implementation of PI in three schools

The qualitative data for the selected schools are presented individually below, and the similarities and differences between them are highlighted. A summary of the highlights is presented in Table 1. *School 1*. When PI was implemented, this school had already been working for a year (2018-2019) with their public health professional and school system navigator (educational consultant) on a similar approach. Thus, the PI presentation to the school team, its needs analysis, and the plan of action had been completed during the previous school year. The school principal, the public health professional, and the school system navigator identified the need to support the reviewing process for the code of conduct (i.e., framework of students’ expected behaviors at school) which was already underway.

**Table 1:** Description of the implementation of PI in three schools.


CASE	SCHOOL CHARACTERISTICS			TARGETED CHILDREN	SCHOOL STAFF INVOLVED			ACTIVITIES CARRIED OUT				
		
	LANGUAGE	SOCIOECONOMIC BACKGROUND INDEX (IN DECILE)	RURALITY		PRINCIPAL	TEACHERS	SUPPORT STAFF	RAISING AWARENESS	MOBILIZING STAKEHOLDERS	COACHING STAFF	ADAPTING TOOLS AND DOCUMENTS	IMPLEMENTING SPECIFIC STRATEGIES

**1**	French	5	Urban	All grade levels	X	X	X	Informal awareness through discussion 1 formal workshop for all staff members*	Meetings with the school team Meeting with parents of 4 years old preschoolers	“Code of conduct” committee “Detention” committee Workshops with lunchtime workers	Revision of the code of conduct according to school’s values Revision of communication cards Implementation of positive bills	Additions of rewarding activities Replacing detention by “quiet time”

**2**	French	10	Rural	Preschool	X	X	–	2 meetings to reflect on practices and students’ needs with kindergarten teachers (3^rd^ meeting canceled*)	Meetings with school teachers Meetings to give systematic feedback to principal	Observation of students in class and at lunchtime Discussions about students’ needs and teachers’ practices Adjustment of teachers’ expectations	No needs identified	Addition of a 2^nd^ special education technician at lunchtime Assigned student’s rank Adjustment of the afternoon schedule according to students’ needs

**3**	English	9	Rural	Preschool and grade 1 and 2	X	X*	X	Workshop #1 for support staff Workshop #1 for teachers*	Joint meetings between Public Health and school psycho-educators	Workshops 2 and 3 with support staff and teachers*	No needs identified	Specific actions to be developed with support staff and teachers (workshop 2 and 3)*


At the beginning of the following school year (2019-2020), PI was quickly setup to support the committee that worked on the simplification and updating of the code of conduct according to the new values identified (safety, responsibility, and respect). In addition, PI guided the school transition in educational practices from a coercive to a positive approach. More specifically, it guided the “detention committee” charged with reviewing and improving disciplinary practices, and also led to the modification of tools and strategies (e.g., revisiting communication cards, creating tools to encourage positive social behaviors, organising reward activities, removing detentions, providing opportunities for quiet time with the teacher).

In addition, the public health professional and school system navigator had regular meetings with school supervisors which helped foster better empathy, kindness, and disciplining with care. They also briefly introduced PI and its concepts in school meetings. It was initially planned to offer PI workshops to all school staff later in the school year (was postponed due to the pandemic). Finally, a parent meeting was facilitated for each of the three 4-year-old preschool groups to introduce them on PI concepts and their use at home.

*School 2*. The school principal prioritized providing support for the two kindergarten teachers as most of the children in these groups displayed significant social and emotional immaturity. This impacted their availability for learning and their ability to function harmoniously in the classroom. Teachers were also expressing that their usual strategies were not sufficient to address the needs of their students. The public health professional and the school system navigator observed the students in their classroom and at lunchtime (i.e., unstructured period of more than 90 minutes). Teachers completed an online questionnaire to identify their respective needs and describe their specific context (e.g., classroom climate, current difficulties, previously used strategies). Three meetings were then scheduled with the teachers to reflect on their practices and their students’ needs. Unfortunately, only two of them took place, as the last one was cancelled because of the pandemic school shutdowns. During these two meetings, teachers were made aware of their students’ specific needs for support, as well as the resources and strategies already in place or available to them. Teachers then adjusted their expectations and interventions with respect to the level of functioning of the children. For example, one teacher planned to reimplement a more structured routine, similar to the one used at the beginning of the school year to better support this group. A special education technician was also added at lunchtime to offer more supervision and support for children who had difficulty staying collected during this unstructured period. Finally, systematic feedback on strategies were carried out with the school principal, leading to continuous mobilization in the school.

*School 3*. The Public health professional first met with the school principal and the school system navigator (psychoeducator) to present PI and discuss the school needs. The school system navigator and the public health professional then held four meetings with another psychoeducator working in this elementary school to further develop the relationship and a common understanding of PI. They also translated documents from French to English and co-constructed workshops for the school team. They agreed to co-facilitate six workshops during the winter and spring (2020). They planned three workshops for support staff (i.e. handicapped student attendants [HSA], special education technician [SET], school supervisors, and daycare educators) and three workshops for teachers (pre-school, grade 1 and grade 2). The first workshop aimed at raising awareness of social and emotional skills’ development as well as introducing PI concepts. The second and third meetings were intended to offer a time to reflect on students’ needs and interventions previously and currently used. The objective was to co-construct specific and personalized ways to promote attachment, empathy, kindness, and healthy discipline with care at the school, based on the needs and issues raised by each group (support staff and teachers). Unfortunately, only the first support staff workshop took place (end of February 2020) before the school shutdown in March 2020. Therefore, no specific strategies were implemented at this school. However, the school system navigator, the public health professional and the school psychoeducator were hoping to review the code of conduct during the next school year in addition to offering the remaining 5 planned workshops.

*Comparisons between schools.:* Two schools (cases #1 and #2) were French-speaking (which is representative of the Eastern Townships region population) and two schools (case #2 and #3) were both rural and had a low socio-economic level. In all schools, the principal and teachers targeted were actively involved in the implementation of PI model. In schools #2 and #3, the plan of action specifically targeted children aged 4 to 8, as prioritized by Public Health, whereas school #1 chose to review their code of conduct, which indirectly benefited all students. Although a workshop for teachers was planned in all three schools, only one school (case #2) was able to offer it before the pandemic shutdown. Schools #1 and #3 prioritized raising support staff awareness. Only one school (school #1) was able to reach parents through an information meeting held at school concerning 4 years old preschoolers. It is worth noting that PI was inspired by a close collaboration established a few years earlier between the school #1, its affiliated school board, and Public Health professional. School #1 has since continued to build on those relationships and the original initiative, with the support of the Public Health professional.

### (3.3) Integration: PI analysis according to Thiam’s conceptual framework for ICC

**Figure 2:**
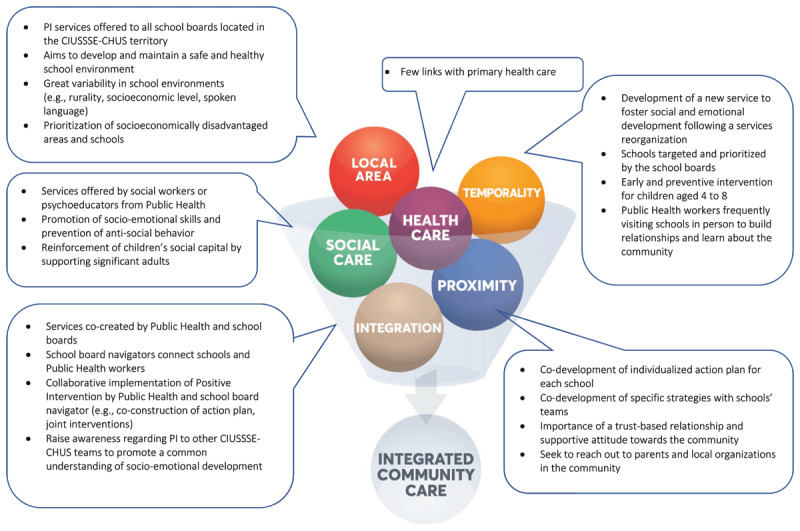
Visual representation of Positive Intervention based on the six components of ICC.

In this section, PI’s main elements are presented according to the six core concepts of the Conceptual Framework for ICC, i.e., temporality, local area, health care, social care, proximity, and integration [28]. As illustrated in Figure 2, PI integrates many components of ICC and leans toward an integrated community social care model. More specifically, through collaborations with schools and implementation of PI, Public Health aims to improve students’ social and emotional skills, as well as to prevent antisocial behaviors. Indeed, PI relies on a strong relational and spatial proximity with intersectoral partners, and more specifically schools. For example, it allows a school board to prioritize which school will first implement PI on their territory, according to the needs identified. This personalisation also allows Public Health professionals to visit schools regularly to build relationships and learn more about its community. Consequently, PI can then foster respect for the intersectoral partner’s temporality when supporting significant adults at different levels of change readiness, and/or schools of different socio-economic levels.

On the other hand, PI is mostly organized around social services and limited partnerships were established with primary care health services after 6 months of implementation. Despite discussions with the managers of the healthcare teams, developing these connections with the Public Health department was not a priority during these first six months. The Public Health department’s priority was the development of strong relationships with their main partners (i.e., schools), then gradually work on building bridges with health services and other partners (e.g., community organizations). Despite only six months of implementation, Public Health professionals were able to carry out a few PI activities with primary care professionals in some local territory. These activities created a common understanding of social and emotional development as well as core concepts of PI.

## (4) Discussion

This paper describes an intervention model that was implemented for 6 months in the Eastern Townships (Quebec, Canada) between September 2019 and March 2020 (school shutdowns due to COVID-19 pandemic). It shows that it is possible to develop and implement an intervention model that promotes social and emotional skills and positive mental health in children, with and for local organizations. PI implementation according to the ICC framework [28] will be discussed, its components analyzed to highlight those that are closely aligned with said framework and suggestions will be made to improve PI’s implementation.

PI can play an important role in the development of students’ social, emotional and behavioral skills, and the collaboration between Public Health and schools may improve positive mental health and prevent antisocial behaviors as well as their associated mid- and long-term consequences [6,8,31,32], which is why its implantation was prioritized in local school settings. PI relies on a strong relational and spatial proximity with their intersectoral partners, with its emphasis on collaboration between public health professionals, school system navigators and schools. It includes adaptations to the stakeholder’s temporality to co-develop a personalized plan of action with strategies tailored to each setting. Although schools with low socio-economic level were given priority in the initial deployment of PI to reduce inequities of care, it is worth mentioning that early and preventive interventions for children aged 4 to 8 to reinforce their social capital is also relevant in schools with a high socio-economic level [21]. In terms of collaborations with local communities, which was initially expected from the logical model, none of the three documented cases were able to achieve this level of implementation during the first six months of PI. However, one school was able to reach out to parents, mainly due to a pre-existing partnership between the health care professional, the school system navigator and the school. It is believed that a strong collaboration with schools will subsequently facilitate the involvement of parents and local communities.

PI currently relies predominantly on social care to promote children’s social and emotional skills and prevent anti-social behaviour with limited connections to the primary health care sector, namely individual psychosocial follow-up for children with adaptation difficulties. Although Public Health professionals initially intended to strengthen relationships with local healthcare teams, this connection was not yet established before the schools’ sudden shutdown. Better integration of Public Health and primary health care services could further improve both health and social outcomes. To do so, stakeholders must consider multiple organizational factors, such as having a clear mandate and reserving time to improve collaboration, valuing each sector, and improving geographic proximity to primary health care professionals [33]. It is also important to consider the change readiness within both sectors to strengthen collaboration, which may take time in some instances [33]. Public Health professionals focused on building a strong collaboration with schools, and then with other stakeholders from the community and primary health care services. However, it has not been possible to fully develop the expected partnerships due to schools shutdown after only six months of implementation.

Although PI does not yet include all characteristics of ICC as described by Thiam et al., it is leaning toward an integrated community social care model. In fact, PI is based on a strong integration at both the management and operational levels. Participation of intersectoral and multidisciplinary teams allows for better integration at the operational level. Those intersectoral and multidisciplinary teams are, as described by Axelsson and Axelsson, “a small group of people, usually from different professions, who are working together across formal organizational boundaries to provide services to a specific group of patients or clients” [22]. Close collaborations with the community, such as the one described in PI between Public Health professionals and school system navigators. allow for a better understanding of their unique reality, leading to the development of a plan of action based on their specific priorities. Knowing that multidisciplinary teams emerging from different organizations (e.g., Public Health and school board) are fragile, a lot of management support is required to ensure sustainability and continuity of these teams [22]. Fortunately for PI, there is a solid collaboration at the management level between the public health organization and school boards. Together these organizations co-constructed PI and both allocated space and released time for professionals to develop and maintain the team relationships. This strong collaboration at the operational and strategic level undeniably streamlines the successful integration of Public Health strategies in schools [6].

This close collaboration between Public Health and school boards is particularly encouraging considering that PI was implemented for only six months and that creating a multidisciplinary team across organizations involves many challenges and takes time [22,34]. This study also demonstrates that it is feasible to implement changes in practices relatively quickly, when partners from different organizational levels are committed to develop services that resemble ICCs. However, since PI was only studied for a short period of time, it is not possible to appreciate its long-term effects. Considering that Publich Health aims to reinstate PI after the pandemic, will they be able to maintain their professional relationships and commitments with the school boards? Will PI be deployed in more communities? What actions will be prioritized by the communities after a two-year shutdown? The COVID-19 pandemic has highlighted, among other things, the need to promote positive mental health, especially in adolescents and young adults. Indeed, this age group is the most at risk for anxiety and depressive symptoms [35]. It might be interesting to explore the implementation of a service like PI, that leans towards ICC, for adolescents and young adults.

This paper is, to our knowledge, the first that describes the implementation of a public health service through an ICC lens. PI does not actually meet all the criteria for ICC, but offers a model for an integrated community social care aiming at fostering healthy social and emotional development. It is therefore interesting to use the Thiam and colleagues’ framework to guide the analysis of PI implementation, as well as to propose avenues for improvements. Since PI implementation has been described in detail in only 3 territories in this study, it is necessary to continue the research to document PI’s social validity and the improvements required to better meet the needs of all partners. In addition, it will be crucial to document the barriers and facilitators of implementing PI, to support other integrated school-public health collaboration initiatives, among other things. Finally, an evaluation of the effects of PI on children and their significant adults would be necessary before reaching the conclusion that it is an effective intervention model.

## (5) Conclusion

This is the first study that explores the implementation of a Public Health service with and for schools to promote the social and emotional development of children aged 4 to 8 years. In brief, PI is a collaborative and personalized intervention model that aims at integrating ICC components and leans towards an integrated community social care model. In fact, PI relies on a strong proximity with school partners and a respect of their each other’s temporality. In this service model, plans of action that promote children’s social and emotional skills are co-developed with and for the school partners, in accordance to the reality of each territory. The level of school-health integration attained after only 6 months of implementation is encouraging for the future, but relationships established with other partners, such as primary health care services, parents and community organizations, were limited during the first 6 months of implementation. More research is needed to thoroughly document the implementation, social validity and effects of such an intervention model by considering the point of view of all stakeholders.
